# Fabrication of a Miniature Multi-Parameter Sensor Chip for Water Quality Assessment

**DOI:** 10.3390/s17010157

**Published:** 2017-01-14

**Authors:** Bo Zhou, Chao Bian, Jianhua Tong, Shanhong Xia

**Affiliations:** 1 State Key Laboratory of Transducer Technology, Institute of Electronics, Chinese Academy of Sciences, Beijing 100190, China; bzhou92@163.com (B.Z.); jhtong@mail.ie.ac.cn (J.T.); shxia@mail.ie.ac.cn (S.X.); 2 School of Electronic, Electrical and Communication Engineering, University of Chinese Academy of Sciences, Beijing 100080, China

**Keywords:** water quality, MEMS, pH electrode, conductivity cell, iridium oxide, electrodeposition

## Abstract

Water contamination is a main inducement of human diseases. It is an important step to monitor the water quality in the water distribution system. Due to the features of large size, high cost, and complicated structure of traditional water determination sensors and devices, it is difficult to realize real-time water monitoring on a large scale. In this paper, we present a multi-parameter sensor chip, which is miniature, low-cost, and robust, to detect the pH, conductivity, and temperature of water simultaneously. The sensor chip was fabricated using micro-electro-mechanical system (MEMS) techniques. Iridium oxide film was electrodeposited as the pH-sensing material. The atomic ratio of Ir(III) to Ir(IV) is about 1.38 according to the X-ray photoelectron spectroscopy (XPS) analysis. The pH sensing electrode showed super-Nernstian response (−67.60 mV/pH) and good linearity (R^2^ = 0.9997), in the range of pH 2.22 to pH 11.81. KCl-agar and epoxy were used as the electrolyte layer and liquid junction for the solid-state reference electrode, respectively, and its potential stability in deionized water was 56 h. The conductivity cell exhibited a linear determination range from 21.43 μS/cm to 1.99 mS/cm, and the electrode constant was 1.566 cm^−1^. Sensitivity of the temperature sensor was 5.46 Ω/°C. The results indicate that the developed sensor chip has potential application in water quality measurements.

## 1. Introduction

Protection of human health is one of the goals of social development, in which water plays an extremely important role. However, the development of industry and agriculture has resulted in water contamination as one of the unintended consequences. Due to the continued deterioration of water sources, the number of water-related diseases and deaths is increasing annually [1]. Therefore, it is intensely demanded to construct monitoring and early warning systems for water quality. Water quality can be assessed from various aspects, through measuring many parameters, including pH, oxidation reduction potential (ORP), conductivity, heavy metal, dissolved oxygen (DO), and so on.

It is cost-consuming and complicated to detect various contaminations separately. Hence, some commercial integrated detection systems have become available [2], which mainly rely on the combination of traditional water sensors, such as bulk pH electrodes and conductivity sensors. Yet, these probes are unsuitable for being deployed on a large scale, because of the relatively high price, complicated structure, and large size. With the progress of microfabrication technologies, some MEMS-based solid sensors for water monitoring have been proposed, such as the Hemin-based DO sensor [3] and carbon nanotube-based free chlorine sensor [4]. Researchers also have paid attention to developing novel integrated sensors that are compact and low-cost for determination of multiple parameters simultaneously. For instance, Zhuiykov et al. [5] have reported an integrated multi-sensor based on screen-printed RuO_2_ thick films to detect the main parameters of water, such as pH, temperature, DO, conductivity, and turbidity. Guijarro et al. [6] have presented a multi-parametric biochip fabricated by MEMS technique, which was capable of simultaneously detecting bioactive pollutants in water. In addition, Wang et al. [7] proposed a microelectrode for simultaneous detection of copper and lead heavy metallic ions.

One goal of this research is to develop a multi-parameter sensor, which has the characteristics of low cost, small size, and convenient operation, for water quality detection. The sensor was expected to be used in a distribution measurement system, where numerous environmental sensors might be emplaced. The proposed sensor in this paper is competent for the detection of pH, conductivity, and temperature in water. The World Health Organization (WHO) recommends that the pH of drinking water should be maintained in the range of 6.5 to 8.5 [8]. Some problems might happen if pH value of the drinking water goes outside the specified range, such as leaching, nitrification, or the presence of microorganisms, which may lead to the gastrointestinal irritation or corrosion of metal pipes [9]. On the other hand, conductivity varies with the dissolved inorganic salts, and, thus, serves as an important factor for indicating ionic contaminations [1]. Temperature is also an important parameter to evaluate water quality and, additionally, it is a dependent factor of the pH value and conductivity. Thus, it is of great significance to develop a miniature and low-cost sensor to measure these three parameters simultaneously.

Traditional glass composite pH electrodes are not suitable for the application in integrated sensors because of the size limitation and brittleness. In order to overcome these problems, researchers have proposed sophisticated alternative pH sensors, such as optical fiber pH sensors [10], ion-selective field-effect transistors [11], solid-state pH sensors [12], and hydrogel film pH sensors [13]. There are extensive studies on potentiometric pH sensors based upon metal oxides, such as RuO_2_ [14], Ta_2_O_5_ [15], SnO_2_ [16], TiO_2_ [12], and IrO_2_ [17], due to the features of mechanical stability, easy integration, and direct electrical response. Among the abundant metal oxides, iridium oxide (IrO_x_) is one of the most promising pH-sensing materials, owing to the fast response, chemical stability, and broad pH sensitive range [17,18].

There are several methods for the preparation of IrOx, including thermal oxidation [19], sputtering [17], sol-gel [20], and electrodeposition [21]. The properties of IrOx may vary with the preparation methods, which affects the performances of IrOx-based pH sensors. For example, IrOx films prepared by sputtering or thermal oxidation are dense and anhydrous, which have a sensitivity close to 60 mV/pH. Whereas, electrochemical methods generate loose and hydrated films, which show uncertain responses in the range of 60 to 90 mV/pH [22]. Some merits and demerits of these fabrication methods have been described in [23]. In spite of the unpredictable potential response and variation in stability for electrodeposited iridium oxide films, electrodeposition is still an attractive way to synthesize IrO_x_ for pH sensors, since there are no requirements for high-temperature operation, vacuum environment, complicated instruments, and expensive targets.

Although many studies have been done on the mechanism of IrOx electrodeposition, there is no consistent explanation to it. Yamanaka [24] and other researchers [25,26,27] supposed that IrOx was deposited on the surface of electrode because the carbon-carbon bonds in the oxalate ligand are oxidized to form CO_2_. However, there are no report directly presenting the decomposition process of mono oxalate complexes. In the Yamanaka solution, Patrick and coworkers [28] found that there were only multinuclear oxyhydroxides and nanoparticles, instead of oxalate complexes, based on which oxalic acid was perceived to merely act as a stabilizing agent. On the other hand, similar IrOx films were prepared using the solutions containing no oxalate ligand [29,30,31], which implied that the deposition may not involve the oxidation of oxalate complex. Recently, researchers seemingly agree with that water is the actual species undergoing oxidization in the deposition of IrOx. Zhao et al. [31] attributed the deposition to the oxidation of the water near the anode. They suggested that the releasing H^+^ ions lower the local pH, which induces the acid condensation of IrO_x_·nH_2_O nanoparticles. This postulation matches that presented in [32], where the authors synthesized the IrO_x_ nanoparticles from a K_2_IrCl_6_ solution, and found that lower pH facilitates the condensation of IrOx nanoparticles. In addition, Heather et al. [29] proposed that a loose polymeric network was precipitated at the surface of electrode, as the result from the oxidization of the water coordinated to Ir(IV).

The reference electrode is an important component in electrochemical measurements. Although some types of standard reference electrodes are efficient, they usually have the features of large size and complicated structure, which limits their applications in integrated sensors [33]. For instance, an Ag/AgCl reference electrode is the most practical, but reducing its scale is difficult because of the unavoidable inner liquid electrolyte and glass tubes. To realize integrated sensors, pseudo-reference electrodes were often integrated on chips using modern fabrication technology. However, such reference electrodes have unstable potentials [34]. The miniaturized liquid junction reference electrode has a better performance than pseudo-reference electrodes, but there are still some challenges, such as sealing a small volume of solution [35] and its maintenance [36]. Thus, it is more applicable for miniature sensors to develop solid-state reference electrodes, where the inner electrolyte is solid [33].

Conductivity sensors are classified into two types: contacting and inductive sensors [37]. Contacting sensors may have two, three, or four electrodes. A four-electrode configuration was perceived more accurate, eliminating the errors caused by polarization and double layers, compared to the conductivity sensors which only have two electrodes [38]. On the other hand, different cell geometries were designed to realize accurate detection of conductivities in various applications. Parallel planar electrodes were usually used to construct miniature conductivity cells [39,40,41], whereas, Heather et al. [42] have introduced a conductivity cell of circular geometry fabricated on the liquid crystal polymer substrate using printed circuit board (PCB)-MEMS technique, a concept that fabricates MEMS through PCB processes. Then the proposed sensor has been successfully employed in coastal salinity measurements [43]. The concentric circular structure defined the directionless profiles, preventing additional calibration. Although the PCB-MEMS technique is cost-efficient and rapid, these two fabrication processes cannot be carried out simultaneously, which might lead to the problems of mass production, and compatibility with the fabrication process of other sensors.

In this paper, we reported the design, fabrication, and experimental results of an integrated sensor. The electrode array, including a four-electrode circular conductivity cell, three-terminal resistive temperature detector (RTD), and substrate electrodes, was fabricated by MEMS techniques. The amorphous hydrous IrOx film was electrodeposited on Pt electrode for pH sensing. A miniaturized reference electrode was formed as reference for pH detection. According to the experimental results, the sensing electrodes on the chip exhibited good performances as intended. The cost of each device is low, since it can be produced in batches. Moreover, the sensor was suitable for various applications, such as remote and distributed measurements, because of the features of small size and mechanical robustness.

## 2. Experimental

### 2.1. Reagents and Instruments

Iridium (IV) chloride (99.95%) was purchased from Alfa Aesar Co. (Haverhill, MA, USA). Hydrogen peroxide (30%), potassium carbonate, oxalic acid, boric acid, phosphoric acid, sulphuric acid, potassium chloride, sodium chloride, and ferricyanide were purchased from Beijing Chemical Works Co. (Beijing, China). All chemicals were analytical grade and used without further purification. All of the water used in the experiments was deionized (18 MΩ·cm) with a Millipore Direct-Q 3 UV (ultraviolet) system (Merck Millipore Co., Billerica, MA, USA). A KCl saturated Ag/AgCl reference electrode was used as a reference, and all potentials were referred to this unless there is an explicit description.

A CHI620e (CH instruments Co., Shanghai, China) electrochemical workstation was used to control the electrochemical experiments. The pH of solutions was verified with a pHS-3C meter (Shanghai INESA Scientific Instrument Co., Shanghai, China). The surface morphology of the IrOx film was investigated using a field emission scanning electron microscopy (FE-SEM) of Merlin Compact (Zeiss Co., Oberkochen, Germany). The chemical composition of the IrOx film was detected by an imaging X-ray photoelectron spectrometer of Axis Ultra (Kratos Analytical Co., Manchester, United Kingdom). The resistance of the RTD was determined using a digital multimeter Agilent 34410A (Agilent Technologies Co., Santa Clara, CA, USA).

### 2.2. Design

**Figure 1:**
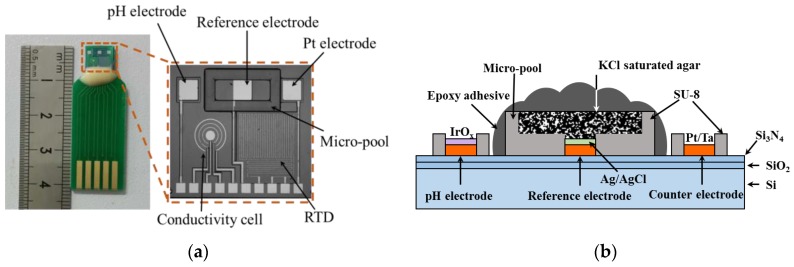
(**a**) Photograph of the multi-parameter sensor chip; and (**b**) a cross-sectional view of the pH sensor; the third (right) electrode acts as the counter-electrode for the electrodeposition of IrOx.

The multi-electrode consisting of a pH sensing electrode, an Ag/AgCl-based solid-state reference electrode, a four-terminal conductivity cell, and a three-end RTD was distributed on the silicon chip, as shown in Figure 1a. Sputtered Pt was used as the materials for conductivity and temperature sensing, because of its good electrical conductivity and temperature characteristic. Additionally, Pt acts as the substrate electrode for pH sensing. The hydrous IrOx film was electrodeposited on the Pt surface for pH sensing. In order to prevent introducing an external macroscopic reference electrode, a solid-state reference electrode was integrated on the chip. Ag/AgCl ink was coated on the Pt electrode. Agar gel containing KCl was used as the inner electrolyte, replacing the liquid junction. Epoxy adhesive keeps the agar from directly contacting to sample solutions. The conductivity cell was designed to be a four-terminal configuration, so that the effect of double electric layer capacitance can be prevented. In the measurement of conductivity, an alternative exciting signal should be applied to the electrodes, which may cause interference with other components. Thereby, our conductivity sensor has a circular geometry, which keeps the electrical field within the defined region from disturbing the other components. Moreover, the RTD has a three-end structure, eliminating the errors caused by lead impedance.

### 2.3. Fabrication of Multi-Parameter Sensor Chip

Standard MEMS techniques were used to fabricate the sensor chip. At first, a p-type silicon wafer was treated by a thermal wet oxidation process and nitration process of low-pressure chemical vapor deposition (LPCVD). Secondly, the substrate metal layer was deposited on the wafer by a direct current (DC) magnetron sputtering method, and then patterned using standard photolithographic and lift-off techniques. The thickness of Pt and Ta (adherent layer) were 300 nm and 30 nm, respectively. A layer of SU-8 negative photoresist was used as an insulation layer, which defines the sensing areas of the electrodes and its pads. Meanwhile, a micro-pool surrounding the reference electrode was formed using SU-8. Specifically, the first SU-8 layer was spin-coated on the wafer and explored with ultraviolet (UV) light. Then, a second layer of SU-8 was immediately coated and explored. Next, one-step development was carried out to shape the insulation layer, combined with the micro-pool. After that, the wafer was diced into chips with a size of 8 mm × 7 mm. Finally, the individual chips were wire-bonded and encapsulated on PCBs.

### 2.4. Fabrication of the Solid State Reference Electrode

The reference electrode was made in three steps. Firstly, Ag/AgCl ink was coated on the Pt electrode, followed by curing at 90 °C for 1 h. Then, an appropriate amount of KCl-saturated agar was sealed in the micro-pool with epoxy adhesive. Finally, we removed the potential gas bubbles in a vacuum desiccator, and then left the electrode at room temperature for about 48 h before coagulation of the epoxy adhesive.

### 2.5. Preparation of pH Sensing Electrode

The IrOx was electrodeposited with a solution introduced by Yamanaka [24]. Briefly, an amount of 0.075 g iridium (IV) chloride was dissolved in 50 mL deionized water with magnetic stirring for 30 min. Then, 0.5 mL hydrogen peroxide (30%) was dripped into the solution with stirring for another 10 min. Subsequently, 0.25 g C_2_H_2_O_4_·H_2_O was added and the solution was kept stirring for 10 min. As the next step, we adjusted the solution pH to 10.5 with potassium carbonate. Finally, the solution was left at room temperature for at least two days until a stable state was reached.

A three-electrode system was employed to electrodeposit the IrOx. The fabricated Pt microelectrode was connected to the electrochemical workstation, acting as working electrode, with an Ag/AgCl (sat. KCl) as the reference electrode, and the third microelectrode on the chip as the counter electrode. After being electrochemically cleaned in 0.05 M H_2_SO_4_, the electrode was immersed in the electrolyte. Deposition was carried out by cyclic voltammetry. After that, the electrode was rinsed with deionized water and dried with fresh air. A cross-sectional view of the pH sensor is showed in Figure 1b.

### 2.6. Measurement Procedure

The properties of IrOx film were investigated with SEM and XPS. Therefore, information can be collected about the morphology and chemical composition of the film. The pH sensing electrode was tested by measuring the open-circuit potential (OCP). Sensitivity of the pH sensing electrode was validated with solutions of different pH levels. Dynamic properties was investigated by a titration test. The performance of the on-chip solid state reference electrode was investigated by cyclic voltammetry (CV) and potentiometry, with a standard Ag/AgCl reference acting as a comparison. The conductivity measurement was conducted with standard buffer solutions of various conductivities. Finally, a water bath experiment was carried out to calibrate the temperature sensor. All of the measurements were performed at room temperature, except for the calibration of the temperature sensor.

## 3. Results and Discussion

### 3.1. Electrodeposition of IrOx

**Figure 2:**
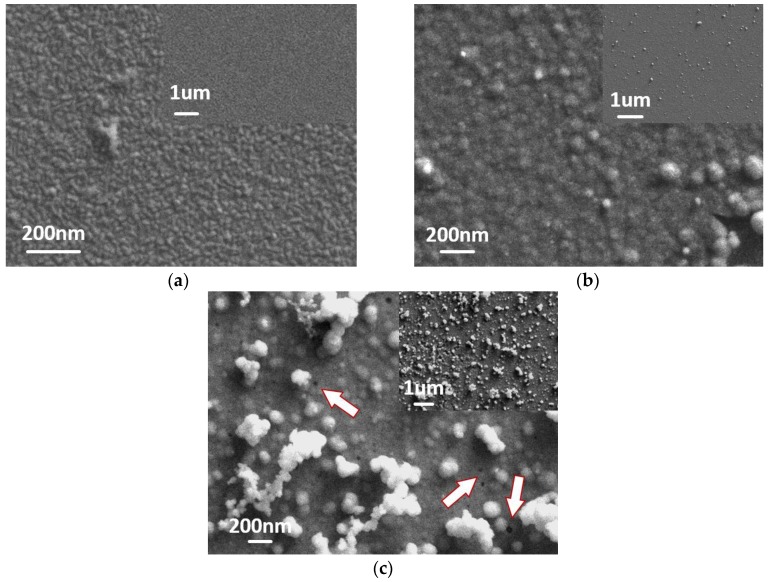
SEM images of the IrOx films deposited in different potential ranges: (**a**) 0–0.6 V; (**b**) 0–0.7 V; and (**c**) 0–0.75 V.

The properties of the IrOx film depend on the composition and morphology of the oxide film, which are mainly dominated by the preparation condition [44]. Thus, potential was investigated as the parameter which could affect the surface morphology of IrOx film, and a SEM was employed to observe morphologies of the films. Different potential ranges (0–0.6 V, 0–0.7 V, and 0–0.75 V) were carried out, while the scan rate (100 mV/s) and the number of voltammetric cycles (100) were kept constant. The SEM images of prepared films were displayed in Figure 2, which illustrate that IrOx nanoparticles have been deposited on the surface of electrode. By comparing the films obtained with different potential conditions, it could be found that a more positive potential induced a lager particle size, and rougher surface. We suppose that a higher potential promotes the process of nucleation of IrO_x_ nanoparticles. Considering the films with rougher surface provide larger number of adsorption centres of pH measurements, a higher anodic potential is desirable. However, if we kept raising the anodic potential limit to 0.75 V, obvious aggregation of IrO_x_ nanoparticles can be found on the surface. Meanwhile, the film showed micropores, some examples being pointed out by the white arrows (Figure 2c). The possible explanation for this phenomenon is that oxygen was released in the process of deposition. When the anodic potential was applied, the water was oxidized to form IrO_x_ nanoparticles, which was reported as an excellent catalyst material for the oxygen revolution reaction [45,46]. With increasing the anodic limits of the potential, the deposited IrO_x_ promoted the abundant release of oxygen, conversely, leading to the micropores. The micropores may lead to the potential drift since the water may diffuse through the pores contacting with the substrate Pt, which affect the potential measurement [47]. Thus, 0–0.7 V was the preferable potential range to synthesize IrOx pH sensing layer. All IrOx films were prepared in this condition in the further experiments.

**Figure 3:**
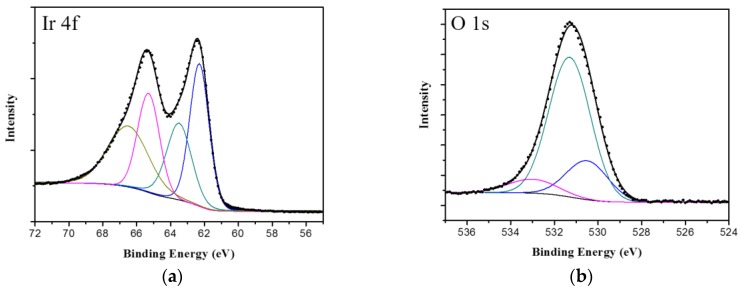
XPS spectra of the surface of iridium oxide film: (**a**) the detailed Ir4f region; and (**b**) the detailed O1s region.

A XPS measurement has been performed to analyze the chemical composition of the film. The spectra were shown in Figure 3. From the high-resolution spectra of the Ir4f region (Figure 3a), it can be found that the binding energy of Ir 4f7/2 and Ir 4f5/2 lines are located at 62.4 eV and 65.4 eV, respectively. According to the curve fitting, which was performed with the constraints described in [48], the ratio of Ir(III) to Ir(IV) was calculated to 1.38. The O 1s signal (Figure 3b) has illustrated the presence of hydroxide and water of hydration on the surface of the film, which implied the prepared film was hydrous.

### 3.2. pH Measurement

**Figure 4:**
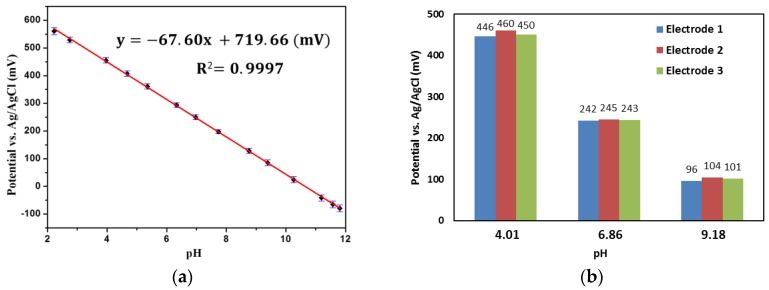
(**a**) Linear potential response of one pH sensing electrode from pH = 2.22 to pH = 11.81; and (**b**) potential responses of three electrodes in standard pH solutions.

The sensitivity of our pH sensing electrode was investigated with different pH standard solutions, which were prepared by mixing 0.2 M NaOH and Britton-Robinson (B-R) buffer solution (0.04 M H_3_BO_3_, 0.04 M H_3_PO_4_, and 0.04 M CH_3_COOH) with various volume ratios. Fourteen solutions with pH levels from 2.22 to 11.81 were used. The electrode was successively dipped in these solutions until the potential reached an equilibrium value. Between the measurements, the electrode was washed with deionized water and dried with fresh air. The test was repeated three times. The potential response was plotted against pH values, as shown in Figure 4a. The pH-sensing electrode exhibited a linear response in the test range, and the sensitivity was calculated to be −67.60 mV/pH.

The pH sensing mechanism of IrOx films is based on the equilibrium between oxides, in which the iridium has different oxidation states [22]. Generally, the mechanism could be described as the following reaction [49]:

(1) $${\mathrm{Ir}}^{\mathrm{IV}}\mathrm{oxide}+xH^{+}+ne^{-}↔{\mathrm{Ir}}^{\mathrm{III}}\;\mathrm{oxide}+yH_{2}O$$

where the values of *n*, *x*, and *y* varied with the preparation method of IrOx, and are essential for determination of the potential response. Thermal oxidation or sputtering methods usually yield anhydrous IrOx films, which have near-Nernstian response with the sensitivity of about 59 mV/pH, because there is an electron per proton transferred in the redox reaction. Nevertheless, a super-Nernstian response, with the sensitivity greater than 59 mV/pH will be obtained when the IrOx films were prepared by electrochemical techniques. This is because the electrochemical method generates hydrated IrOx layers, in which case the transferred electrons are less than the involved hydrogen ions. The sensitivity greater than 59 mV/pH indicates that the IrOx prepared in our work was hydrate. Similar results were demonstrated in [21], where the IrOx showed super-Nernstian response as well.

We tested different electrodes in pH 4.01, 6.86, and 9.18 solutions, and the response was showed in Figure 4b. The relative standard deviations (RSD) of the response for these electrodes were 1.60%, 0.63%, and 4.03%, indicating a good reproducibility of the electrode. It was suggested that the reproducibility and reliability of the hydrated IrOx-based pH sensor is associated with the hydrate level [21,50] and oxidation state [22] of the film. The test result implies that the films have a similar hydrate level and oxidation state.

**Figure 5:**
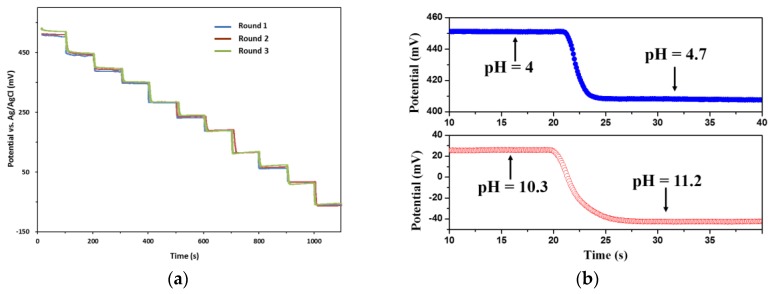
(**a**) The real-time potintial response of the pH sensing electrode; and (**b**) the response time measured from pH = 4.03 to pH = 4.70 (**upper**) and from pH = 10.28 to pH = 11.19 (**lower**).

A titration test was carried out to investigate the dynamic property of the pH sensing electrode. In the experiment, the pH sensing electrode was placed in a beaker containing 20 mL B-R buffer solution. Then, 2 mL NaOH (0.2 M) solution was dropped into the beaker every 100 s for adjustment of the pH level. Magnetic stirring was employed to accelerate the progress towards the equilibrium state. A commercial pH meter calibrated with standard buffer solutions (pH = 4.01, 6.86, 9.18) was employed to verify the pH values. During the test, the OCP of the pH sensing electrode was recorded by an electrochemical analyser. The potential responses of three measurement cycles were shown in Figure 5a, illustrating the potential changed quickly with the titration event. However, there are some potential differences at the same pH levels found in the graph, which is a common phenomenon for most metal oxide-based pH sensing electrodes [51]. In our case, the maximum difference in the detected pH levels was 16 mV, which indicates that our pH sensing electrode has good repeatability due to the acceptable IrOx film quality.

The time required to reach 90% of the equilibrium value was defined as the response time, which is an important indicator of dynamic property. In order to know whether the sensing film has the same quick response in different environments, the response times were measured in acid and alkaline conditions, respectively. Figure 5b shows the potential response changing over two transition steps, from which we can suggest that the response time was less than 7 s. The quick response facilitates the reduction of power consumption for detection system. It was also found that a longer time was needed to reach equilibrium in the alkaline condition. This is probably due to the lower concentration change of hydrogen ions before and after titration in an alkaline environment.

### 3.3. Characteristics of the Solid State Reference Electrode

**Figure 6:**
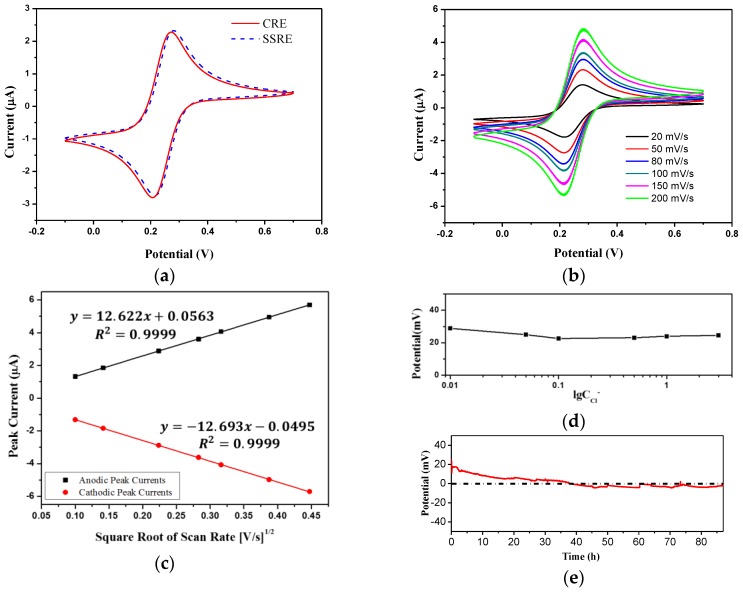
Electrochemical characteristics of the SSRE: (**a**) CVs for SSRE (----) and CRE (**—**) in 2 mM ferricyanide at 50 mV/s; (**b**) CVs for SSRE at various scan rates: 20, 50, 80, 100, 150, and 200 mV/s; (**c**) variations of the anodic (■) and cathodic (●) peak currents vs. the square root of the scan rates; (**d**) potentials of the SSRE at KCl concentrations ranging from 0 to 3 mol/L; and (**e**) the stability of the SSRE measured in deionized water over 86 h.

The electrochemical characteristics of the solid state reference electrode (SSRE) were investigated by cyclic voltammetry (CV) and potentiometry, with a commercial Ag/AgCl reference electrode (CRE) acting as the comparison. The CV measurements were carried out using 2 mM ferricyanide in 0.1 M KCl as a redox couple. The SSRE or CRE was dipped in the solutions as the reference electrode, an on-chip Pt electrode as the working electrode, and a commercial Pt electrode as the counter electrode. The CV curves of SSRE and CRE, obtained in the potential range from −0.1 to 0.7 V, at the scan rate of 50 mV/s, are almost coincident as shown in Figure 6a. When a series of scan rates (20, 50, 80, 100, 150, and 200 mV/s) were applied, the CVs of SSRE were recorded and shown in Figure 6b. The anodic and cathodic peak current is linearly proportional to the square root of the scan rate (Figure 6c), which matches the theory of the diffusion-controlled process.

KCl solutions with different concentrations were used to measure the independence to the Cl^−^ concentration of the SSRE. The freshly prepared electrode was dipped in the solutions and the OCP versus the CRE was recorded after an equilibrium state was reached. Figure 6d shows the potential curve plotted against the logarithm of the KCl concentration, illustrating that the potential of the SSRE is around 24 mV and scarcely influenced by the Cl^−^ concentration. This fixed potential vs. CRE is probably due to the unequal concentration of Cl^−^ for the SSRE and the CRE. The potential stability of the SSRE was evaluated by recording the OCP vs. the CRE in deionized water. Over an 86-hour measurement, the potential drift rate is 0.3 mV/h as shown in Figure 6e. In the first 30 h the potential decreased faster at a rate of 0.6 mV/h, and then remained stable, from which the stability of our SSRE was found to be at least 56 h.

The microscopic voids existing in the epoxy offer an ionic connection between the inner KCl-agar and the sample solutions, on the other hand, the epoxy efficiently protected the inner ${\mathrm{Cl}}^{-}$ from fast fluxing into the sample solutions. Table 1 illustrates the performance comparison of our SSRE and some other SSREs for pH sensing in the literature. The stability of the Ag/AgCl/GO reference electrode is 26 days in 3 M KCl solution with test intervals [52]. A LBL-PE/npPt reference electrode exhibited a stability of 50 h in 0.1 M PBS [53], and a screen-printed Ag/AgCl/KCl-glass SSRE showed a stability of 4 h in deionized water [54], while our SSRE exhibited a longer stability in deionized water. Additionally, the fabrication method of our SSRE is very simple, without any specific apparatus needed, and it can be used and stored freely, as the cured epoxy has good adhesiveness and high mechanical strength. Therefore, the SSRE in this work is suitable for the application in water quality monitoring, where intermittent measurement strategy is used.

### 3.4. Conductivity Measurement

**Figure 7:**
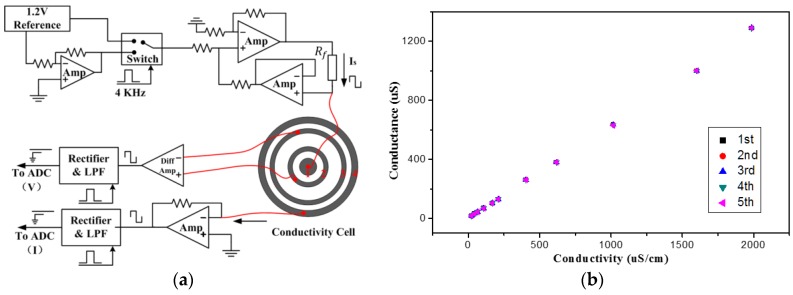
(**a**) Schematic of the trans-impedance measurement circuit; and (**b**) linear calibrition curve of the conductivity cell.

A four-electrode conductivity cell is like a four-terminal precision resistor. The conductivity of water was determined by detecting the trans-impedance of the cell. A supporting circuit was constructed providing an appropriate signal to drive the cell. When a DC signal was used to excite the transducer, the interfacial capacitance between electrodes and water, which is known as an electrolytic double-layer capacitance, may cause the impedance errors, resulting in a smaller readout [55]. Thus, an alternative current with a sufficiently low amplitude is preferable. It was proved that the trans-impedance is a function of the frequency, and there is a clear conductivity-sensitive interval for medium frequencies [41,56]. In our case, an alternating square wave was applied to excite the cell, and 4 kHz was selected as the optimal operating frequency. Figure 7a shows the diagram of the supporting circuit for the trans-impedance measurement. The amplitude of the drive current was controlled by adjusting the resistance of the reference resistor ($R_{f}$). The trans-impedance of the cell was determined by detecting the excitation current passed over the two drive electrodes (ring 1 and 4) and the potential drop between the sensing electrodes (rings 2 and 3). The conversion from the measured conductance to the specific conductivity is derived from Equation (2):

(2) C=k×I/E

where *I* is the excitation current through the sensor, *E* is the voltage drop between the sensing electrodes, *k* is the factor determined by the cell itself and called the cell constant.

The conductivity cell was calibrated with the standard solutions prepared with the recipe described in [57]. The test was repeated five times. The measured conductance was plotted against the conductivity of the solution (Figure 7b), from which the cell constant was calculated to be 1.566 cm^−1^. The maximum relative deviation was 3.37%, which shows that the conductivity sensor has good repeatability. Meanwhile, the linear range of the conductivity sensor was from 21.43 μS/cm to 1.99 mS/cm, which covers the conductivities of usual drinking water [1].

### 3.5. Temperature Measurement

**Figure 8:**
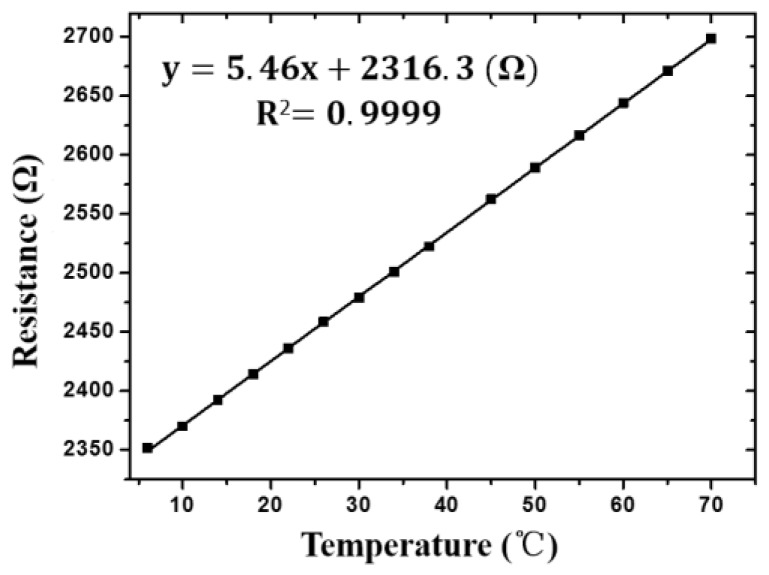
The linear relationship between resistance of RTD and temperature.

Motivated by the need of accurate temperature measurement, the RTD was designed as a three-terminal structure to prevent the errors caused by lead impedance. The temperature sensor was calibrated against a commercial temperature probe using a water bath. The resistance of the RTD was measured with the multi-meter in the test. The relationship of the resistance of RTD and temperature is shown in Figure 8. The sensitivity (5.46 Ω/°C) and good linearity (R^2^ = 0.9999) indicate that the RTD could be used to detect temperature accurately, compensating for the thermal errors for the measurements of pH and conductivity.

## 4. Conclusions

In this paper, we proposed a multi-parameter sensor chip, on which three different sensors were distributed. The chip size was approximately 7 mm × 8 mm. The circular shape for the conductivity sensor was designed to prevent the bipolar square wave from disturbing the other electrodes. Hydrous IrOx film was electrodeposited on the surface of the Pt electrode as the pH sensing material, coupling with a miniaturized solid-state reference electrode. The on-chip sensors exhibited good performance as intended. The pH sensor showed a sensitivity of −67.60 mV/pH, and had a short response time. The conductivity cell had a sensitivity of 1.566 cm^−1^, which is suitable for the detection of drinking water. The temperature sensor had a high sensitivity of 5.46 Ω/°C. The integrated sensing chip could be manufactured in batches, which makes it economical and suitable for application in portable or online detection systems of water quality. Further work will focus on the optimization and fusion of multiple transducers, as well as the construction of the water quality monitoring system. Lifetime is the most important issue for on-site measurement, so we will mainly aim for the extension of the sensor lifetime. A low-power consumption circuit will be developed as the data acquisition node, so that the sensor can be used in water quality monitoring.

## Figures and Tables

**Table 1:** Comparison of the performance of some solid-state reference electrodes for pH sensing.

Electrode Type	Fabrication Techenique	Test Solution	Test Mode	Stability	Literature
Ag/AgCl/GO	Sputtering, chemical chlorination, and drop casting	3 M KCl	Intermittent (2 or 3 days’ interval)	26 days	2015 [52]
LBL-PE/npPt	Electrodeposition, photo polymerization	0.1 M PBS	Continuous	50 h	2011 [53]
Ag/AgCl/KCl-glass	Screen printing	Deionized water	Continuous	4 h	2014 [54]
Ag/AgCl/KCl-agar/epoxy	Drop casting	Deionized water	Continuous	56 h	This work
